# Induction of CD73 prevents death after emergency open aortic surgery for a ruptured abdominal aortic aneurysm: a randomized, double-blind, placebo-controlled study

**DOI:** 10.1038/s41598-022-05771-1

**Published:** 2022-02-03

**Authors:** Harri Hakovirta, Juho Jalkanen, Eija Saimanen, Tiia Kukkonen, Pekka Romsi, Velipekka Suominen, Leena Vikatmaa, Mika Valtonen, Matti K. Karvonen, Maarit Venermo, INFORAAA Study Group, Harri Hakovirta, Juho Jalkanen, Eija Saimanen, Tiia Kukkonen, Pekka Romsi, Velipekka Suominen, Leena Vikatmaa, Mika Valtonen, Matti K. Karvonen, Maarit Venermo, Veikko Nikulainen, Jan-Erik Wickström, Kimmo Kaskinoro, Pirkka Vikatmaa, Ilkka Kantonen, Riikka Tulamo, Ilkka Uurto, Damir Vakhitov, Matti Pokela, Arjaleena Ilo, Mika Mäenpää, Miikka Frant, Toni Pihlaja, Juha Koskenkari, Jyrki Virkkunen, Raili Laru-Sompa, Anni Pulkkinen, Hannu Savolainen, Antti Mäkelä, Seppo Hovilehto, Asser Aavik, Jüri Lieberg, Geidi Rajaste, Jaak Kals, Andrus Metsa, Toomas Ellervee, Heli Järve, Silver Sarapuu, Kadri Tamme, Linas Velicka, Martynas Laukaitis, Tadas Lenkutis, Nerijus Misonis, Ingrida Asekiene, Arminas Skrebunas, Gintautas Kekstas

**Affiliations:** 1 Turku University, Kiinanmyllynkatu 4-8, 20520 Turku, Finland; 2 Satasairaala, Pori, Finland; 3 Department of Vascular Surgery, Turku University Hospital, Turku, Finland; 4 Faron Pharmaceuticals Ltd., Turku, Finland; 5 Department of Surgery, South Karelia Central Hospital, Lappeenranta, Finland; 6 Department of Vascular Surgery, Hospital Nova of Central Finland, Jyvaskyla, Finland; 7 Department of Vascular Surgery, Oulu University Hospital, Oulu, Finland; 8 Department of Vascular Surgery, Tampere University Hospital, Tampere, Finland; 9 Department of Anesthesiology, Intensive Care, and Pain Medicine, Helsinki University Hospital, Helsinki, Finland; 10 Department of Perioperative Services, Intensive Care and Pain Management, Turku University Hospital, Turku, Finland; 11 Department of Vascular Surgery, University of Helsinki and Helsinki University Hospital, Helsinki, Finland; 12 Department of Intensive Care, Oulu University Hospital, Oulu, Finland; 13 Department of Intensive Care, Hospital Nova of Central Finland, Jyvaskyla, Finland; 14 Department of Intensive Care, South Carelia Central Hospital, Lappeenranta, Finland; 15 Department of Vascular Surgery, Tartu University Hospital, Tartu, Estonia; 16 Department of Cardiac, Thoracic and Vascular Surgery, Hospital of Lithuanian University of Health Sciences Kauno Klinikos, Kaunas, Lithuania; 17 Centre of Reconstructive Vascular and Endovascular Surgery, Vilnius University Hospital Santoros Klinikos, Vilna, Lithuania

**Keywords:** Aneurysm, Surgery

## Abstract

Mortality remains high after emergency open surgery for a ruptured abdominal aortic aneurysm (RAAA). The aim of the present study was to assess, if intravenous (IV) Interferon (IFN) beta-1a improve survival after surgery by up-regulating Cluster of differentiation (CD73). This is a multi-center phase II double-blind, 2:1 randomized, parallel group comparison of the efficacy and safety of IV IFN beta-1a vs. placebo for the prevention of death after open surgery for an infra-renal RAAA. All study patients presented a confirmed infra-renal RAAA, survived the primary emergency surgery and were treated with IFN beta-1a (10 μg) or matching placebo for 6 days after surgery. Major exclusion criteria included fatal hemorrhagic shock, chronic renal replacement therapy, diagnosed liver cirrhosis, severe congestive heart failure, advanced malignant disease, primary attempt of endovascular aortic repair (EVAR), and per-operative suprarenal clamping over 30 min. Main outcome measure was all-cause mortality at day 30 (D30) from initial emergency aortic reconstruction. The study was pre-maturely stopped due to a reported drug-drug interaction and was left under-powered. Out of 40 randomized patients 38 were included in the outcome analyses (27 IFN beta-1a and 11 placebo). There was no statistically significant difference between treatment groups at baseline except more open-abdomen and intestinal ischemia was present in the IFN beta-1a arm. D30 all-cause mortality was 22.2% (6/27) in the IFN beta-1a arm and 18.2% (2/11) in the placebo arm (OR 1.30; 95% CI 0.21–8.19). The most common adverse event relating to the IFN beta-1a was pyrexia (20.7% in the IFN beta-1a arm vs. 9.1% in the placebo arm). Patients with high level of serum CD73 associated with survival (P = 0.001) whereas the use of glucocorticoids and the presence of IFN beta-1a neutralizing antibodies associated with a poor CD73 response and survival. The initial aim of the trial, if postoperative INF beta-1a treatment results on better RAAA survival, could not be demonstrated. Nonetheless the anticipated target mechanism up-regulation of CD73 was associated with 100% survival. According to present results the INF beta-1a induced up-regulation of serum CD73 was blocked with both use of glucocorticoids and serum IFN beta-1a neutralizing antibodies. The study was pre-maturely stopped due to interim analysis after a study concerning the use if IV IFN beta-1a in ARDS suggested that the concomitant use of glucocorticoids and IFN beta-1a block the CD73 induction.

**Trial registration:** ClinicalTrials.gov NCT03119701. Registered 19/04/2017 (retrospectively registered).

## Introduction

A ruptured abdominal aortic aneurysm (RAAA) is a medical emergency. The only lifesaving treatment is immediate aortic surgery. Half of RAAA patients die before reaching the hospital, and despite successful surgery another 30–40% of these patients die post-surgery in intensive care due to multi-organ failure (MOF)^1^. The cause of MOF is systemic inflammatory response brought forth by inflammatory mediators that are released from stressed and dying cells. Although operative techniques and intensive care have developed during the past decades, little change has occurred in post-operative mortality for open surgery of RAAA. The only measure to improve the post-operative survival seem to be centralization of open aortic repair (OAR) to high volume centers^2,3^. Endovascular aortic repair (EVAR), which does not require the cross-clamping of the aorta and does not produce surgical trauma to the extent as open-repair, has emerged as new surgical option and to provide better RAAA outcome also in low volume centers^2,4^. This could be result of minor surgical trauma and hence post-operative systemic inflammatory response is milder after EVAR compared to open repair, which could lead to less MOF and better survival^5^.

Based on earlier literature CD73 has an important role in vascular health and the progression of atherosclerosis in experimental animal models in an age dependent manner^6–8^. As in critically ill patients CD73 is considered a key organ protective endothelial molecule in states of hypoxia and inflammation^9,10^. In vascular beds CD73 is the rate limiting enzyme in producing anti-inflammatory adenosine from pro-inflammatory adenosine triphosphate (ATP), which is released from stressed and dying cells^11^. CD73 is up regulated on the endothelium by hypoxia itself, or by type I interferons (IFNs)^12^. IFN beta-1a is known to induce CD73 and attenuate inflammation in critical care settings if given intravenously (IV)^13^. IV IFN beta-1a is still an investigational medicinal product but considered a viable treatment option for adult respiratory distress syndrome (ARDS), Middle East respiratory syndrome (MERS) and coronavirus (COVID-19)^14^. In the current trial we set out to test the ability of IV IFN beta-1a induced CD73 to prevent MOF and death after emergency aortic reconstruction for RAAA in a randomized and placebo-controlled setting. The study was pre-maturely stopped after an interim analysis, that was performed because another study concerning the use if IV IFN beta-1a in ARDS reported that the concomitant use of glucocorticoids inhibits IFN beta-1a from up-regulating CD73^15,16^. Glucocorticoids are widely used in intensive care due to their profound anti-inflammatory and hemodynamic properties, especially in septic shock, although they have shown contradictive results in patient outcomes in a number of trials^17^. More recently in the era of COVID-19 literature suggest using type I IFNs and glucocorticoids in a sequenced manner, respectively^18^.

## Methods

### Study design

This was a multicentre, phase II, 2:1 randomised, double-blind, parallel group comparison of the efficacy and safety of IV IFN beta-1a compared to placebo in the prevention of death in adult patients surviving emergency open surgery for an infra-renal RAAA.

The study was designed to enrol patients who presented with a RAAA diagnosed by ultrasound or computed tomography (CT) scan in the emergency room. All ruptures were confirmed (= retroperitoneal haematoma) in operation, and only confirmed ruptures were included. Patients underwent screening when a diagnosis of RAAA had been made. Before entering the study, patients were to be assessed to ensure that all eligibility criteria were met. Study specific procedures were performed only after obtaining written informed consent from the patient itself, if applicable in the emergency department, or from the next of kin.

Patients meeting all the inclusion and none of the exclusion criteria at screening were randomly assigned to treatment with IFN beta-1a or placebo in the randomisation ratio of 2:1 (IFN beta-1a: placebo). The treatment assigned to each patient was determined according to a computer-generated randomisation list within an electronic case report form (eCRF). Following randomisation, after successful surgery, the first dose of the study drug was administered at arrival to the intensive care unit (ICU). No longer than 4 h could pass after arriving to the ICU before administering the study treatment. This allowed some time for assessing the stability of the patient and seeking consent from next of kin, without un-necessarily prolonging the beginning of the treatment. Consecutively the patients were treated daily with 10 μg of IFN beta-1a or matching placebo as an IV bolus followed by a 5 ml flush of sterile saline for up to 6 days every 24 h (± 2 h) starting from the first dose. Treatment could be stopped earlier if the patient improved and was discharged from the ICU. The main analysis and reporting used D30 follow-up.

The trial was conducted according to Good Clinical Practice and ICH Guidelines according to the Declaration of Helsinki. The trial was first approved by the institutional review board Ethics Committee of the Hospital District of South-West Finland and of then at each participating site (identifier ETMK:62/1800/2014), and informed consent was obtained before any study procedure in accordance with local processes. The present study was registered on ClinicalTrials.gov NCT03119701 on 19/04/2017. The study was monitored by an independent Data Monitoring Committee that reviewed on-going safety data in an unblinded manner.

### Eligibility criteria

Patients older than 18 years with emergency open infra-renal RAAA surgery were included for the study. The infra-renal RAAA was confirmed by imaging or during emergency surgery. Temporary clamping above the renal arteries to control the surgical situation did not lead to exclusion unless total suprarenal clamping time exceeded 30 min. This was because kidney function was assessed as an endpoint and prolonged clamping could imbalance study groups.

Major exclusion criteria were irreversible hemorrhagic shock, unconsciousness at arrival or during screening, chronic renal replacement therapy, diagnosed liver cirrhosis, severe congestive heart failure defined as NYHA class IV, advanced malignant disease, EVAR first attempt, known hypersensitivity to natural or recombinant IFN or on-going treatment with IFN alpha or beta-1a.

### Outcome measures

The primary outcome was all-cause mortality at day 30 (D30). Key secondary endpoints included organ failure-free days at D30, ventilator free days at D30, days on hemodialysis, length of ICU stay, length of hospital stay, and all-cause mortality at D90. For the definition and managements of adverse events (AE) please see Supplement 1. Adverse events were recorded between the informed consent and D30.

The Myxovirus Resistance Protein A (MxA) is and clinically applicable biomarker for interferon beta-1a activation^19^. Therefore, both the biological activity and effect of the study drug were analyzed by detecting Myxovirus Resistance Protein A (MxA) and CD73 levels were measured from collected serum samples during study days 1–13 with enzyme-linked immunosorbent assays as described previously Myxovirus resistance protein A^15^. The former is a specific biomarker of IFN beta-1a activity, the latter is the molecular target of IV IFN beta-1a. To further analyze the possible confounding factors for the effect of INF beta-1a the neutralizing antibodies for against INF beta-1a were analyzed as described in Supplement 1 and the possible post-operative use of glucocorticoids was analyzed since our recent data suggest that glucocorticoids might inhibit the INF beta-1a induced CD73 upregulation^16,20^. Please see online Supplement 1 for further details on sample collection and methos for NABS, MaX and CD73 analyses.

### Statistical analysis

A sample size of 129 eligible patients for the primary efficacy analysis, with a 2:1 ratio to active (86) and placebo (43), was planned to detect the difference of 22% between the two treatment groups with 80% power using 2-sided test with 0.05 significance level. Because of the planned interim analyses, the calculations assume that 2 sequential tests are made, and the O’Brien–Fleming spending function is used to determine the test boundaries. The sample size calculations were to be re-evaluated after interim analyses at 40 and 84 evaluable patients. Logistic regression model was used for the analyses of observed mortality rates in the two treatment groups. Data for the primary efficacy endpoint was presented for key, pre-defined subgroups of patients: patients with concomitant use of glucocorticoids with study drug, CD73 responders (twofold increase from baseline value), and actual treated patients receiving at least two doses of study drug, i.e. Per Protocol Set (PPS). To assess the association of CD73 response with glucocorticoid use, average and maximum CD73 values from days 3–13, i.e. after at least two doses of study drug, between overlapping glucocorticoid use and non-use were tested with t-test (using Satterthwaite t-test in case the variances were unequal). The same test was used to assess the association of CD73 values between patients alive and not alive at D30. Further exploratory analyses concerning CD73 are described along with Supplemental Results.

Descriptive statistics were provided for all treatment-emergent adverse events (TEAEs). TEAEs were summarised using frequency and patient-based tables. All safety analyses were conducted for the entire enrolled patient population, i.e. Full Analyses Set (FAS). All statistical tests were performed as two-sided and at a significance level of 0.05.

### Ethics approval and consent to participate

The trial was approved by the institutional review board of each participating site, and informed consent was obtained before any study procedure in accordance with local processes.

### Consent for publication

The present article is original and bases on data that has not been published or communicated to a society or meeting at any form. All authors have given their consent for publication.

## Results

### Study participants

**Figure 1:**
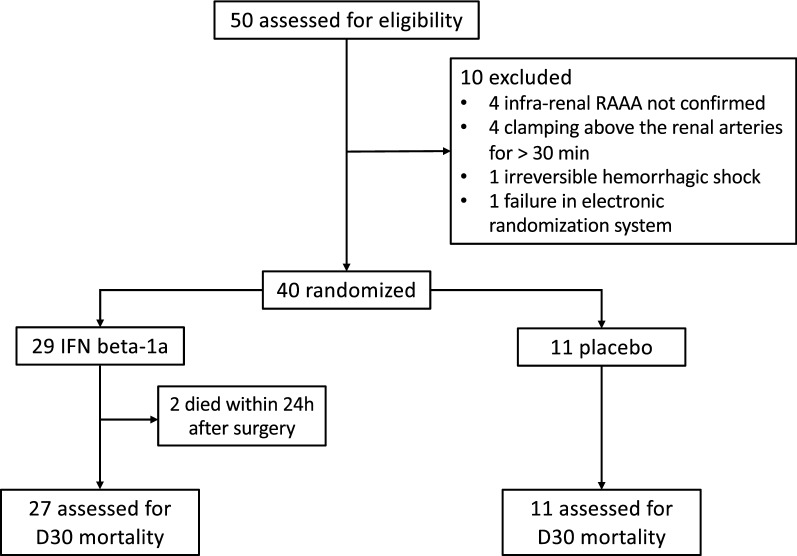
CONSORT diagram.

Patients were enrolled between February 2017 and September 2019 at 9 sites in Finland and the Baltic countries. A total of 50 patients were screened, out of which 40 patients fulfilled all inclusion criteria and were randomly assigned and received treatment in the study at the time of the interim analysis: 29 patients were allocated into the IFN beta-1a group and 11 patients into the placebo group. A total of 30 (75.0%) patients completed 6-day dosing (22 [75.9%] and 8 [72.7%] patients in the IFN beta-1a and placebo groups, respectively). All 40 (100.0%) randomised patients were included in the FAS population and 38 (95.0%) in the PPS population evaluating drug efficacy. The two patients that were excluded from the PPS population died within 24 h after surgery, which does not allow sufficient time for the study drug to work and be evaluated for efficacy. These patients were evaluated for safety and detailed description of these patients can be found in the online Supplement. No patient discontinued the study or was lost for follow-up (Fig. 1).

The overall mean age of the study subjects was 73.6 (SD, 8.95) years and similar in both active treatment and placebo groups. The percentage of male patients enrolled in the study was higher compared with female patients. All the patients were Caucasian. Overall, the demographic characteristics were similar in both treatment groups (Table 1). The median duration of the index surgery for RAAA was 181 min, and similar between the treatment groups. The median total volume substitution, total blood substitution and total crystalloid substitution for patients were rather similar between the treatment groups, although there was a tendency of more fluids and platelets used in the active arm. In approximately half of the study patients (16 [42.1%]) the abdomen was left open at the index surgery: more frequently in the active group (13 [48.1%]) compared to the placebo group (3 [27.3%]).

**Table 1:** Baseline demographics and procedural characteristics.

	IFN beta-1a N = 27	Placebo N = 11	p value
**Demographics**			
Age	73.4 (SD, 9.6)	73.9 (SD, 7.6)	0.87
Sex (male)	85%	100%	0.30
Hypertension	16 (59%)	5 (46%)	0.49
Atrial fibrillation	4 (15%)	4 (36%)	0.20
Coronary artery disease	4 (15%)	1 (9%)	1.00
Hypercholesterolemia	9 (33%)	3 (27%)	1.00
Diabetes	5 (19%)	2 (18%)	1.00
COPD	5 (19%)	2 (18%)	1.00
Asthma	3 (11%)	1 (9%)	1.00
**Major surgical features**			
Duration of surgery (min)	186 (SD, 76)	169 (SD, 55)	0.49
RBC substitution (mL)	1573 (SD, 946)	1378 (SD, 972)	0.57
Platelet substitution (mL)	357 (SD, 603)	173 (SD, 212)	0.18
Total volume substitution (mL)	7159 (SD, 4133)	6294 (SD, 1938)	0.39
Left renal vein ligated	3 (11%)	0 (0%)	0.54
Clamp above the renal arteries	2 (7%)	1 (9%)	1.00
Open abdomen	13 (48%)	3 (27%)	0.30

*COPD* chronic obstructive pulmonary disease, *RBC* red blood cell.

### Outcomes and biomarkers

At this data cut-off 38 patients (27 in the active arm and 11 in the placebo arm) were included in the efficacy analyses. Two patients were excluded from efficacy analyses, because they died within 24 h after arriving in the ICU post-surgery, and this is too early of IFN beta-1a to induce CD73. Both excluded patients had been allocated to the IFN beta-1a treatment group. The Data Monitoring Committee carefully examined these two cases. Both patients were deeply hypotensive and anuric already from surgery due to underlying illnesses and massive bleeding. Overall treatment was ceased within 24 h of surgery. Both deaths were attributed to be a cause of the underlying condition and not the study drug (please see Supplement for further details).

**Figure 2:**
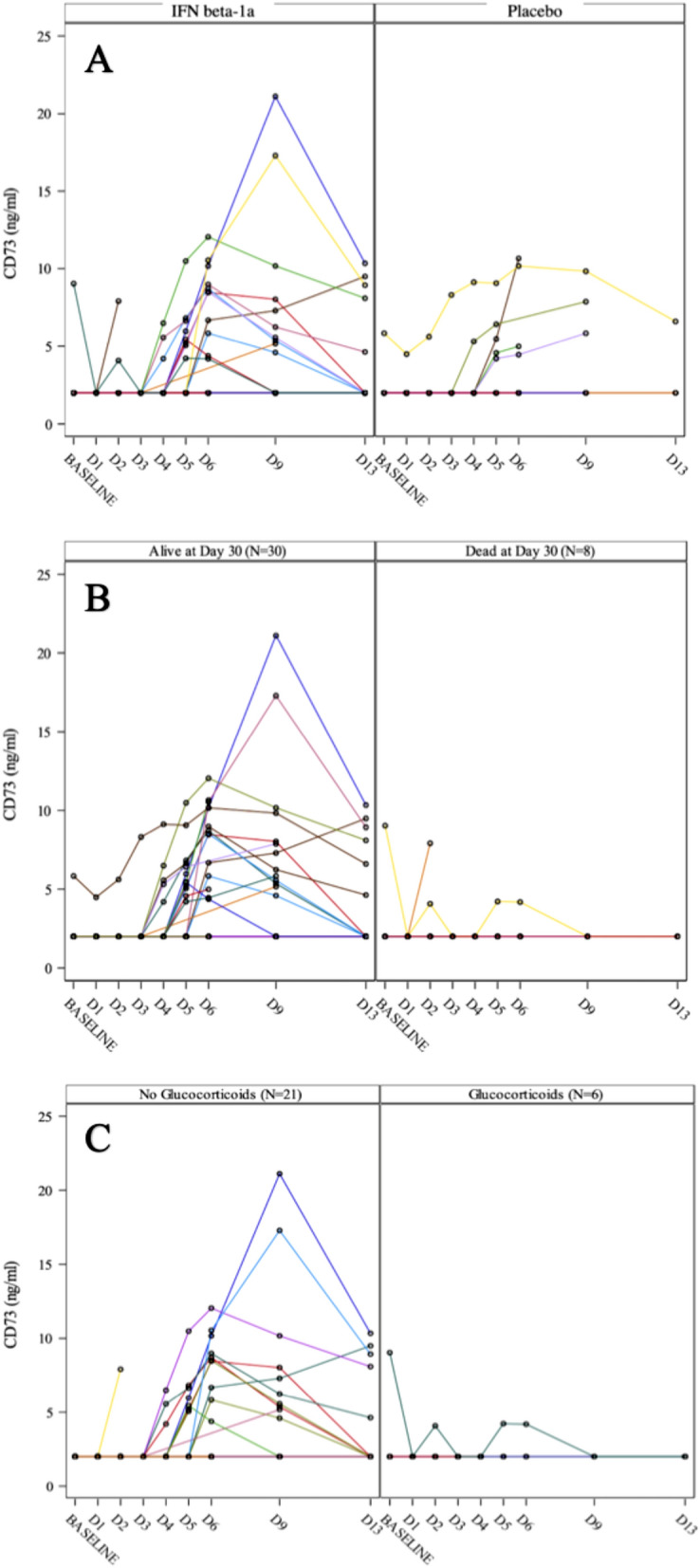
Individual CD73 responses (**A**) in the active vs. placebo arms (no significant difference), (**B**) in patients that survived vs. patients that died by D30 (i.e. all study patients), mean max CD73 achieved between groups 6.09 ng/mL (SD, 4.95 ng/mL) correspondingly for patient without glucocorticoids vs. 2.37 ng/mL (SD, 0.90 ng/mL) (Satterthwaite t-test, P < 0.001), and (**C**) in patients that received vs. did not receive overlapping glucocorticoids with IV IFN beta-1a (i.e. active arm only), mean max CD73 achieved between groups 6.52 ng/mL (SD, 5.37 ng/mL) for patient without glucocorticoids vs. with glucocorticoids 2.37 ng/mL (SD, 0.91 ng/mL) (Satterthwaite t-test, P = 0.0026).

There was no statistical difference in the primary endpoint between study groups in the entire safety population (FAS, N = 40) or efficacy population (PPS, N = 38) (Table 2). All-cause mortality at D30 was 22.2% (6 of 27 patients) in the interferon beta-1a (FP-1201-lyo) group and 18.2% (2 of 11 patients) in the placebo group (OR 1.30; 95% CI 0.21–8.19). No secondary efficacy endpoint showed a significant difference between study groups either. However, subgroup analyses revealed that there was a trend of increased mortality in patients that received glucocorticoids during the study treatment. This was seen in both active and placebo arms (Table 2). D30 mortality was 19% in patients not receiving overlapping glucocorticoids with the study drug compared to 33.3% in patients receiving glucocorticoids in the active arm. In similar fashion D30 mortality was 14.3% vs 25% depending on glucocorticoid use in the placebo arm. Further biomarker analyses measuring serum CD73 could not demonstrate significant difference between study groups (Fig. 2A) but showed that an elevation in CD73 significantly associated with survival at D30 (P = 0.001) (Fig. 2B). This was seen in the entire patient population (Table 2). Overlapping use of glucocorticoids with the study drug was associated with no CD73 response. This was statistically significant especially in the active arm (P = 0.002) (Fig. 2C). Details on the use glucocorticoids together with IFN beta-1a are presented in Supplemental Table S1. Supplemental Figures S1 and S2 illustrates the head-to-head comparison of the entire population divided into CD73 responders and non-responders, which show the survival benefit and improvement in SOFA score following a CD73 response.

**Table 2:** Primary outcome for the patients with INF beta 1a treatment vs. placebo, CD73 responders vs. non-responders and postoperative glucococorticoid treatment vs. no glucocorticoid.

	At D30	INF beta-1a	Placebo	Total
		% (n)	% (n)	% (n)
All	Survival	78.8% (21)	81.8% (9)	78.9% (30)
CD73 response+	Survival	100% (8)	100% (1)	100% (9)
CD73 response−	Survival	31.6% (6)	20% (2)	27.6% (8)
Glucorticoids	Survival	66.7% (4)	75% (3)	70% (7)
No glucocorticoids	Survival	81% (17)	85.7% (6)	82.1% (23)

### IFN beta-1a neutralizing antibodies

**Figure 3:**
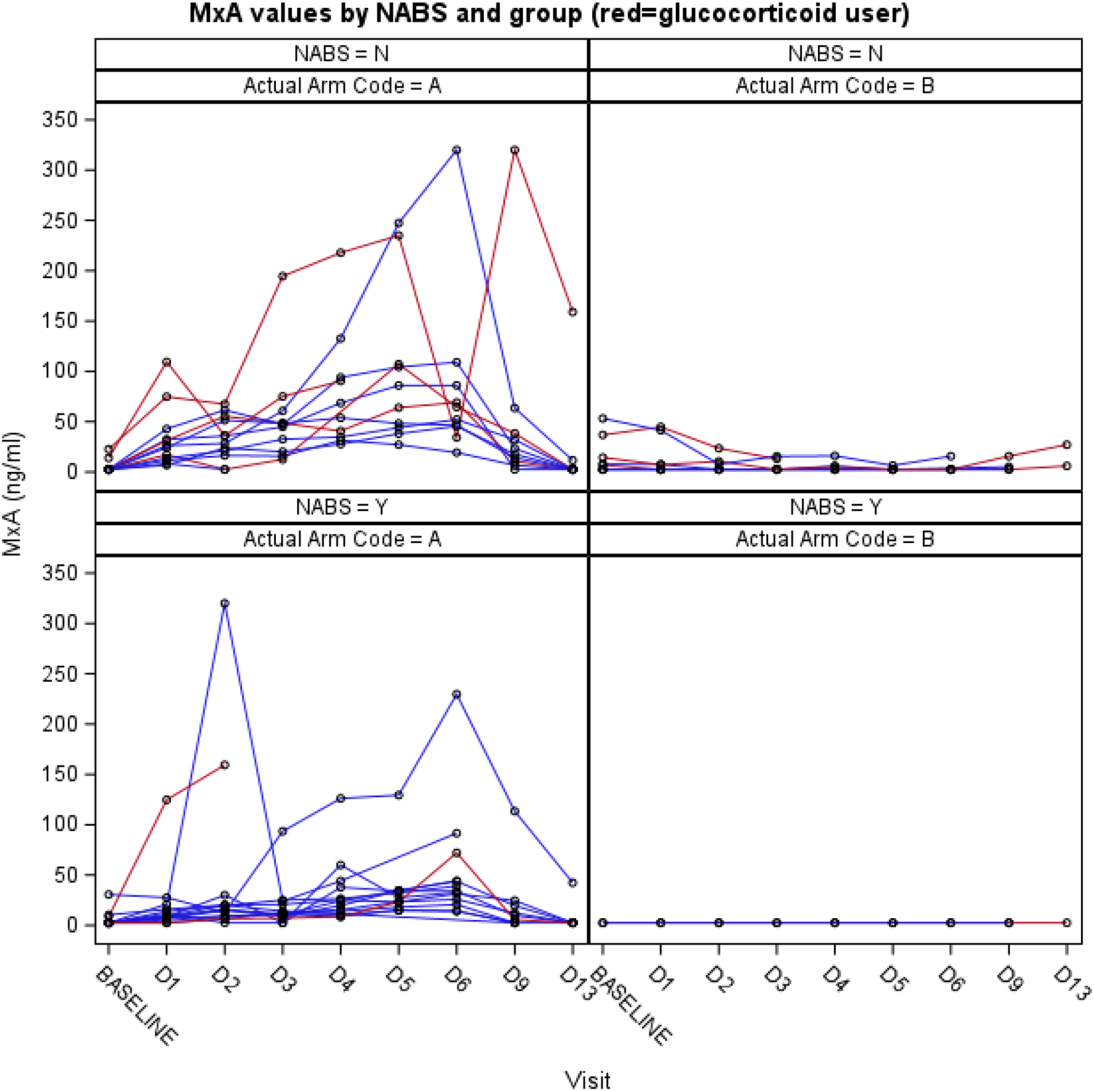
Myxovirus resistance protein A (MxA) levels in both treatment groups (A = IFN beta-1a group; B = placebo group) according to the presence of IFN beta-1a neutralizing antibodies (NABS) (Y = yes, elevated levels; N = elevated levels not detected).

**Figure 4:**
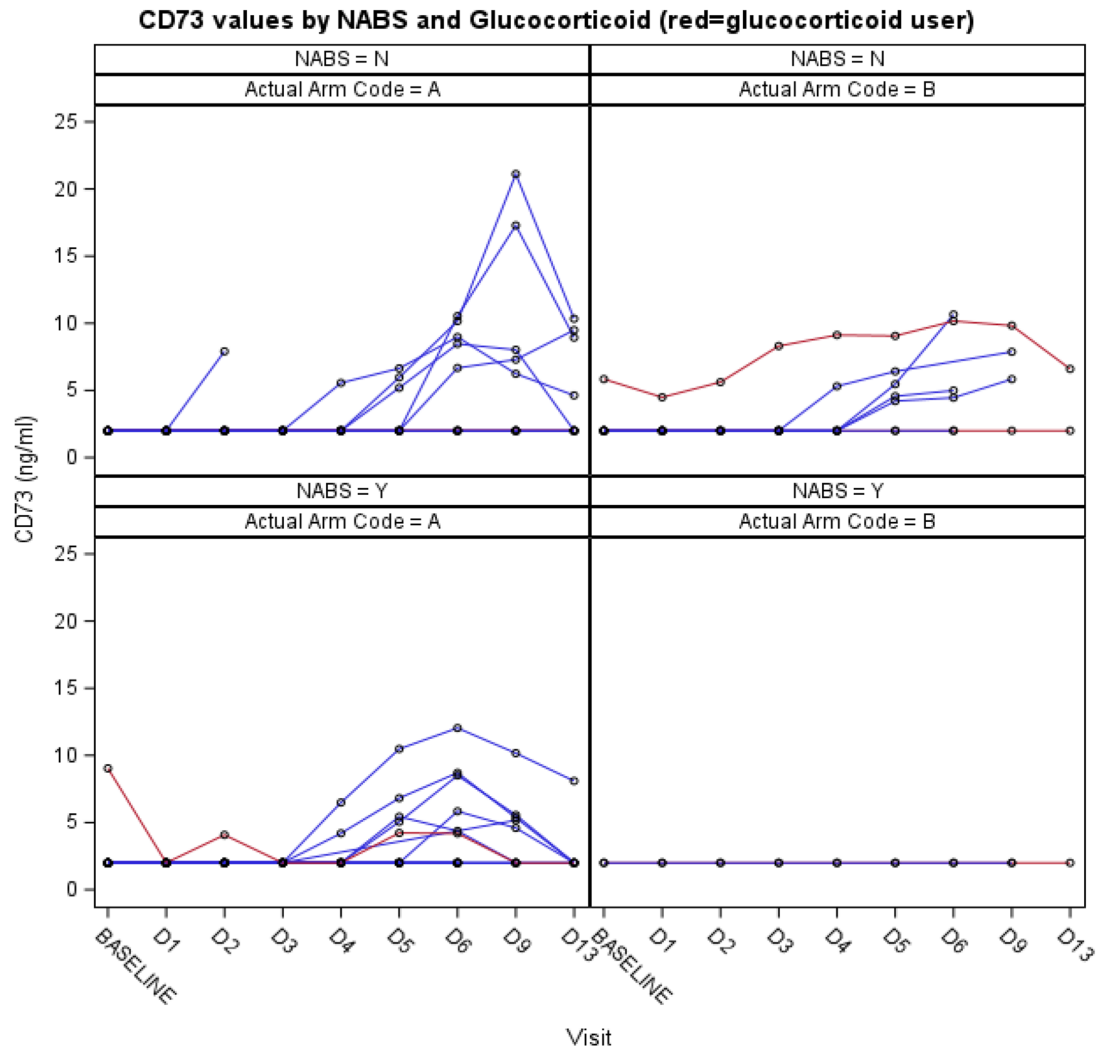
Cluster of differentiation (CD73) values in both treatment groups (A = IFN beta-1a group; B = placebo group) according to the presence of IFN beta-1a neutralizing antibodies (NABS) (Y = yes, elevated levels; N = elevated levels not detected).

The interaction of glucocorticoids with IFN beta-1a did not fully seem to explain why certain individual did not respond to the IFN beta-1a treatment. As a part of the studies safety analyses, the development of IFN beta-1a neutralizing antibodies (NABS) was monitored. We did not see any increase in the development IFN beta-1a NABS during treatment, but 45% of patient had elevated titres of NABS at baseline, which was un-expected. These titres normalized by day 30, i.e. IFN beta-1a treatment did not produce NABS. Elevated baseline titres of NABS associated significantly with lower levels of both CD73 (P = 0.04) and MxA (P = 0.02) during the study in multivariate model of repeated measurement (RM ANCOVA). In the multivariable model the presence of NABS was strongest and only statistically significant variable that affected CD73 levels during the observation period (baseline to day 13). Figures 3 and 4. Overall, in the study population D30 mortality was 14% in patient without NABS and 29% in patients that had elevated levels of NABS.

### Safety

For the safety analyses, 40 patients (29 patients in the active arm and 11 patients in the placebo arm) were exposed to study drug, and 75.9% and 72.7% of patients in the FP-1201-lyo and placebo group, respectively, completed 6 days of study treatment. There were no differences between groups in treatment exposure duration.

Altogether, 36 (90.0%) patients experienced treatment-emergent adverse events (TEAEs) during the study. Overall, the number and percentage of patients experiencing at least 1 TEAE were similar in the 2 treatment groups (27 [93.1%] patients in the IFN beta-1a group compared with 9 [81.8%] patients in the placebo group). Pyrexia and atrial fibrillation were the most common TEAEs, with a different incidence in both treatment groups: pyrexia occurred at a higher frequency in the IFN beta-1a group (6 [20.7%] patients vs. 1 [9.1%] patient in the placebo group), whereas new onset atrial fibrillation occurred at a higher frequency in the placebo group (3 [27.3%] patients in the placebo group and 5 [17.2%] patients in the IFN beta-1a group). Serious TEAEs were reported in 16 (40%) of patients. The number and percentage of patients experiencing at least 1 serious TEAE were higher in the IFN beta-1a group compared with the placebo group (13 [44.8%] patients in the active group compared with 3 [27.3%] patients in the placebo group). None of these differences were statistically significant. The most common TEAEs concerned gastrointestinal ischemia and perforations, which formed the clear majority (7 out of 13) of the reported serious TEAEs in the active arm. Out of these 4/7 had open abdomen from surgery and 5/7 of these patients died. A clear feature was, that 6/7 of these patients with intestinal ischemia were patients that did not have a CD73 response. In the placebo arm 1 patient had intestinal ischemia and died. TEAEs leading to discontinuation of the study drug occurred in 6 (15%) patients overall, of which 5 [17.2%] were from the IFN beta-1a compared to 1 [9.1%] in the placebo (Table 3). Liver enzyme increases were the most common TEAE leading to discontinuation, which occurred in the IFN beta-1a group only (2 [5.0%] patients). In summary, 9 (22.5%) patients died during the study, plus an additional 2 patients shortly after surgery. Overall, the incidence of fatal outcomes was similar in the treatment groups and no clear safety concern was observed.

**Table 3:** Treatment-emergent adverse events leading to discontinuation of study drug in the safety population.

System organ class	Reported term	IFN beta-1a (N = 29) N (%)	Placebo (N = 11) N (%)
Any TEAE	Total	5 (17.2)	1 (9.1)
Cardiac disorders	Total	1 (3.4)	0 (0.0)
	Myocardial ischaemia	1 (3.4)	0 (0.0)
Gastrointestinal disorders	Total	0 (0.0)	1 (9.1)
	Intestinal ischaemia	0 (0.0)	1 (9.1)
General disorders	Total	2 (6.9)	0 (0.0)
	Multiple organ dysfunction syndrome	1 (3.4)	0 (0.0)
	Pyrexia	1 (3.4)	0 (0.0)
Laboratory investigations	Total	3 (10.3)	0 (0.0)
	Hepatic enzyme increased	2 (6.9)	0 (0.0)
	Liver function test abnormal	1 (3.4)	0 (0.0)

## Discussion

In the INFORAAA trial we randomly assigned infra-renal RAAA patients to receive either 10 μg of IV IFN beta-1a or matching placebo for up to 6 days during their ICU stay after surviving emergency aortic reconstruction. The aim was to prevent death by INF-beta-1a induced activation of CD73. All patients with high CD73 levels after emergency RAAA surgery survived, but all treated with INF beta-1a did not have the anticipated INF beta-1a induced CD73 response.

Therefore, present RCT failed to demonstrate the positive effect of INF beta-1a on post-operative RAAA survival. Both overlapping use of glucocorticoids during the intervention period and the existence IFN beta-1a neutralizing antibodies appear to have prevented the desired IFN beta-1a induction of CD73, and thus, preventing the possible positive effect of INF beta-1a on survival. The same drug-drug interaction has recently been reported in an ARDS study^15^ and shown to happen in primary human pulmonary endothelial cells because glucocorticoids block the formation of transcription factor binding to the interferon response element in the nucleus^16^. Also recently, a high level IFN neutralizing antibodies have been reported to associate with severe COVID-19^21^, a condition leading to ARDS and MOF. It has been postulated that the presence of IFN neutralizing antibodies, which increase with age, are a cause that leads to severe COVID-19^21,22^. However, here in a blood sample taken after a ruptured aorta we find a surprisingly high rate of IFN neutralizing antibodies, in 45% of patients, which are in an inflammatory storm. The question is, are this IFN neutralizing antibodies the cause or result of the witnessed inflammatory and vascular crisis.

A zero-mortality was observed in patients that had a CD73 response. Even though the study population was relatively small, such a clear-cut observation highlights CD73’s role as an organ protective and possibly lifesaving molecule in ischemic conditions^23,24^. A notable observation, which conquers to current knowledge, is that hypoxia itself is a strong inducer of CD73^25,26^. This was observed also in the placebo arm where elevation in CD73 values were seen in similar fashion as in the IFN beta-1a arm 3–4 days after surgery. However, it did not happen in similar intensity as in the IFN beta-1a group. Inducing CD73 with IFN beta-1a or hypoxia requires time, because it happens through response elements in the gene encoding for CD73, which then leads to protein transcription and expression of the protein^26^. One can consider this as a form high altitude acclimatization, which also takes time. CD73 has been shown to increase at altitude or hypoxemic conditions^27^, and to be crucial to prevent hypoxia induced vascular leakage^9^. Remote ischemic pre-condition, i.e. introducing intermittent hypoxic periods to a torniquet limb, also induces systemic CD73 and has been sought to be organ protective when done before coronary artery bypass grafting (CABG)^28^. So far large clinical trials investigating ischemic pre-conditioning in CABG have been disappointing^29^, but then again ischemic periods to the limb have mainly been applied during anesthesia induction, which does not allow sufficient time for induction of CD73 prior to surgery. Pre-operative statin treatment has been shown to be beneficial prior to CABG and other major cardiovascular surgery^30^. Amongst their many effects, statins are also known to stabilize endothelial CD73^31^. From dose finding studies with intravenous IFN beta-1a, it is known that induction of prominent pharmacodynamic effect happens within 2 to 3 daily doses if measured using MxA from circulating lymphocytes, i.e. the anti-viral effect of IFN beta-1a^13^. Concerning induction of CD73, it is likely that induction happens in a similar time frame than with MxA, but quantifying CD73 elevations is more complex. In vitro a similar time frame for CD73 induction has been shown on human endothelial cells^13^. Direct measurement of CD73 expression on the endothelium is not possible in patients, and measuring endothelium shed serum CD73 is a surrogate marker reflecting endothelial happenings in a delayed fashion. Ideally, patients would benefit the most from early administration of IV IFN beta-1a before the ischemic insult and inflammatory responses. However, this would have to be explored in a different setting than RAAA. For RAAA patients any mean to improve the turnover of extracellular ATP into adenosine is likely to benefit patients after they have survived the immediate crisis and surgery^27^.

The IFN beta-1a treatment was well tolerated and did not show any clear safety concern in this extremely ill and hazardous patient population. IFN beta-1a treatment was especially associated with pyrexia, which is a known adverse effect of type I IFNs^15^. Liver failure could be a concern in this extremely ill patient population, but only a small number of cases with increased liver enzymes were observed. Intestinal ischemia and perforations are rather common in RAAA patients^32^, and there was a higher but not significant incidence of mesenteric ischemia in the active arm. CD73 has been shown to be a key molecule to protect also from gastrointestinal ischemia^25^ and most patients with gastrointestinal ischemia (6/7) patients had no CD73 response. Overall, the active arm had more surgical challenges as indicated by longer procedure duration, volume and platelet substitution, and incidence of left renal vein ligation, together with not being able to close the abdomen.

### Limitations

Investigating a pharmaceutical intervention in a population of RAAA patients is extremely challenging. Firstly, the incidence of RAAA is low and variable, which makes enrollment a challenge. This study was slow in recruiting, partly due to the increasing number of EVARs performed for RAAA instead of open surgery. Open surgery instead of EVAR was chosen because the systemic inflammatory response is significantly bigger after open surgery^28^. Secondly, patients are very volatile and suffer a higher number of complications, especially surgical complications, which makes the study outcome measures sensitive to challenges faced in the index surgery. Thirdly, investigators and healthcare professionals looking after these patients operate under highly stressful and demanding conditions, which makes overall running and monitoring the trial challenging. The study was designed to manage all these variables to the best possible extent to capture a coherent and comparable population that could still receive the study drug and possibly benefit from it in comparison to placebo. Thus, we left out impending ruptures, thoracoabdominal aneurysms, or patients that were not likely to survive, because they would add significant variability to the outcome and imbalance between groups. We also chose not to administer the study drug before surgery, for example in the emergency department, because subsequent blood loss and surgical features could have altered the drug effect even further and led to a higher degree of early dropouts and un-certainty on the drug effect. Nonetheless, the biggest limitation of this study to draw any definitive conclusions concerning its original aim is that it is significantly under-powered due to pre-mature interruption.

## Conclusions

Contrary to the initial hypothesis IV IFN beta-1a did not prevent post-operative RAAA mortality. Further studies are required to draw definitive conclusion as the clinical trial had to be stopped and did not reach the expected numbers of participants. Future trial should take into account potential interaction with glucocorticoids and interferon beta-1a neutralizing antibodies. Nonetheless the anticipated target mechanism up-regulation of CD73 was significantly associated with high survival and is a potential site of drug action to improve post-operative RAAA survival.

## Supplementary Information

Supplementary Information.

## Data Availability

The study data is available on request 4Pharma Ltd. (Turku, Finland) Timo Huttunen. info@4pharma.com.

## Abbreviations

ARDS: Adult respiratory distress syndrome
ATP: Adenosine triphosphate
CD73: Cluster of differentiation
COPD: Chronic obstructive pulmonary disease
COVID-19: Coronavirus disease 2019
CT: Computed tomography
D30: Day 30
D90: Day 90
eCRF: Electronic case report form
EVAR: Endovascular aortic repair
FAS: Full analyses set
FP-1201-lyo: Interferon beta-1a
ICU: Intensive care unit
IFN: Interferon
IV: Intravenous
MERS: Middle east respiratory syndrome
MOF: Multi-organ failure
MxA: Myxovirus resistance protein A
NYHA: New York Heart Association
PPS: Per protocol set
RAAA: Ruptured abdominal aortic aneurysm
RBC: Red blood cell
TEAE: Treatment-emergent adverse events
