# Supplementary material for: Induction of CD73 prevents death after emergency open aortic surgery for a ruptured abdominal aortic aneurysm: a randomized, double-blind, placebo-controlled study
Source: Sci Rep. 2022 Feb 3;12:1839. doi: 10.1038/s41598-022-05771-1 (PMC8813993; doi:10.1038/s41598-022-05771-1)
Supplement: Supplementary file 1 — Supplementary Information. [file 41598_2022_5771_MOESM1_ESM.docx]

**SUPPLEMENT 1**

**Induction of CD73 prevents death after emergency open aortic surgery for a ruptured abdominal aortic aneurysm – a randomized, double-blind, placebo-controlled study**

Harri Hakovirta MD, PhD^1,2,3^; Juho Jalkanen MD, PhD^4^; Eija Saimanen MD, PhD^5^; Tiia Kukkonen MD, PhD^6^; Pekka Romsi MD, PhD^7^; Velipekka Suominen MD, PhD^8^; Leena Vikatmaa MD, PhD^9^, Mika Valtonen MD, PhD^10^; Matti K. Karvonen MD, PhD^4^; Maarit Venermo MD, PhD^11^; and INFORAAA Study Group

***Definition and management adverse events (AE)***

It is recognised that the patient population in the ICU experience a number of common aberrations in laboratory values, signs and symptoms due to the severity of their underlying disease and the impact of standard therapies. These will not necessarily constitute an AE unless they require significant intervention, lead to discontinuation of blinded study drug or are considered to be of concern in the Investigator’s clinical judgement.

Each AE will be assessed by the Investigator with regard to the following categories:

Seriousness
An serious adverse event (SAE) is defined as any untoward medical occurrence that at any dose:

- Results in death
- Is life-threatening. This means that the patient is at risk of death at the time of the event. It does not mean that the event hypothetically might have caused death if it were more severe
- Requires inpatient hospitalisation or prolongation of existing hospitalisation
- Results in persistent or significant disability or incapacity
- Is a congenital anomaly or birth defect
- Is an important medical event that may not be immediately life-threatening or result in death or hospitalisation but that may jeopardise the patient or require intervention to prevent one of the above outcomes. Examples of such events are intensive treatment in an emergency room or at home for allergic bronchospasm; blood dyscrasias or convulsions that do not result in hospitalisation; or development of drug dependency or drug abuse

“At any dose” does not imply that the patient is receiving study treatment at the time of the event. Study drug doses may have been given during treatment cycles or interrupted temporarily prior to the onset of the SAE, but may have contributed to the event.

Intensity

Classical reporting of mild, moderate and severe AEs making a reference to the patient’s functional status is difficult for a randomised study in critically ill patients. Patients enrolled in this study will primarily be mechanically ventilated and comatose due to their underlying condition and/or the drugs they are prescribed for sedation and analgesia in the ICU. Therefore, the classical approach to AE reporting, which requires patient communication and evaluation of the impact on functioning will be adapted to the ICU environment. The Investigator will be responsible for the assessment of severity, using the categories of mild, moderate or severe to describe each AE as:

- **Mild:** Does not interfere with patient’s usual function
- **Moderate:** Interferes to some extent with patient’s usual function
- **Severe:** Interferes significantly with patient’s usual function

Note the distinction between serious and severe AEs. **Severe** is a measure of intensity whereas an event must meet one of the criteria for serious events listed in previously to be considered **serious**; thus, a **severe** reaction is not necessarily a **serious** reaction. For example, a headache may be severe in intensity, but would not be classified as serious unless it met one of the criteria for serious events listed previously

Causality

The Investigator assess the causality/relationship between the study drug and the AE and record that assessment in the eCRF. Causality will be assessed as:

- **Not related:** AE is obviously explained by another cause*; or* the time of occurrence of AE is not reasonably related to administration of the study drug
- **Possibly related:** Study drug administration and AE occurrence are reasonably related in time*; and* AE is explained equally well by causes other than study drug
- **Probably related:** Study drug administration and the occurrence of the AE are reasonably related in time*; and* the AE is more likely explained by exposure to study drug than by other mechanisms

The most likely cause of an AE (e.g., disease under treatment, concomitant disease, concomitant medication, other) will be indicated in the eCRF with details of the concomitant disease or medication or other cause.

Clinical Laboratory Adverse Event

Abnormal laboratory findings (e.g., biochemistry, haematology, urinalysis) or other abnormal assessments (e.g., vital signs) that are judged by the Investigator as clinically significant will, if certain requirements are met, be recorded as AEs or SAEs. Clinically significant abnormal laboratory findings or other abnormal assessments that meet the definition of an AE or SAE and are detected during the study, or are present at baseline and significantly worsen following the start of the study, will be reported as AEs or SAEs. However, clinically significant abnormal laboratory findings or other abnormal assessments that are associated with the disease being studied (unless judged by the Investigator as more severe than expected for the patient’s condition), or that are present or detected at the start of the study and do not worsen, will not be reported as AEs or SAEs.

The Investigator will exercise their medical and scientific judgment in deciding whether an abnormal laboratory finding or other abnormal assessment is clinically significant.

*Recording Adverse Events*

AE reporting extend from signing of informed consent. AEs occurring after D30 should be reported to the Sponsor by the Investigator if the Investigator considers there is a causal relationship with the study drug. However, all deaths will be recorded and reported as SAEs throughout the study (up until D90).

All AE reports should contain a brief description of the event, date and time of onset, date and time of resolution, intensity, treatment required, relationship to study drug, action taken with the study drug, outcome, and whether the event is classified as serious.

Recording a diagnosis (when possible) is preferred to record a list of associated signs and symptoms. However, if a diagnosis is known but there are associated signs or symptoms not generally attributed to the diagnosis, the diagnosis and each sign or symptom must be recorded separately.

*Reporting Serious Adverse Events*

According to applicable European Union regulations and requirements, an SAE must be reported to the Sponsor from the trial site as soon as possible **within 24 hours** of becoming aware of the SAE. A medically qualified person at the trial site identified on the Delegation of Authority Log with this responsibility must assess the SAE. Any member of the clinical trial site staff can assist in reporting an initial SAE. The Principal Investigator or delegated sub-investigators are responsible for the SAE reporting procedures at the site during the trial, and must always sign-off on each SAE even if other site staff have reported the event on behalf of the investigators. A delegation log at each trial site will clearly show delegation of responsibilities regarding SAE reporting.

The Investigator and the Sponsor (or Sponsor’s designated agent) will review each SAE report and the seriousness and the causal relationship of the event to study treatment will be evaluated. In addition, the Sponsor (or Sponsor’s designated agent) will evaluate the expectedness according to the reference document (Investigator Brochure). Based on the Investigator and Sponsor’s assessment of the event, a decision will be made concerning the need for further action.

If consensus on the assessment cannot be reached between the parties (e.g., Investigator and Sponsor/Sponsor’s delegate), all opinions will be provided in the Council for International Organizations of Medical Sciences Form I report and reporting to the CA and IEC should be based on the highest degree of causality provided.

Details for reporting SUSARs can be found in Section bellow.

All SAEs will be recorded that occur between signing of informed consent and D30. Events occurring after D30 and coming to the attention of the Investigator should be reported only if they are considered in the opinion of the Investigator to be causally related to the investigational drug. However, all deaths up to D90 will be reported as SAEs.

All SAEs occurring as described above, must be reported **within 24 hours** by email or fax.

The minimum information required for an initial report is:

- Details of person sending the report (i.e., name and address of Investigator)
- Patient identification details (screening/randomisation number, age, sex, NOT patient name)
- Protocol number
- Description of SAE
- Causality assessment

*Follow-up of Adverse Events*

All AEs experienced by a patient, irrespective of the suspected causality, will be monitored until: the AE has resolved; any abnormal laboratory values have returned to baseline or stabilised at a level acceptable to the Investigator and Medical Monitor; there is a satisfactory explanation for the changes observed; the patient is lost to follow-up; or the patient has died.

*Suspected Unexpected Serious Adverse Reactions*

Any AE that is serious, associated with the use of the study drug, and unexpected (SUSAR) has additional reporting requirements, as described below.

- If the SUSAR is fatal or life threatening, associated with use of the study drug and unexpected, regulatory authorities and IECs must be notified **within 7 calendar days** after the Sponsor learns of the event. Additional follow-up information (cause of death, autopsy report and hospital report) should be reported **within an additional 8 days** (15 days total).
- If the SUSAR is not fatal or life threatening but is otherwise serious, associated with the use of the study drug and unexpected, regulatory authorities and IECs must be notified **within 15 calendar days** after the Sponsor learns of the event.

The Sponsor will notify the Investigators in a timely fashion of relevant information about SUSARs that could adversely affect the safety of patients. Follow-up information may be submitted if necessary.

The Sponsor will also provide annual safety updates to the regulatory authorities and IECs responsible for the study. These updates will include information on SUSARs and other relevant safety findings.

**Details from the Adverse Event Reports on two patients that were excluded from efficacy analyses**

Patient 1

Patient 1, an 80-year-old male diagnosed with ruptured abdominal aortic aneurysm (RAAA),was randomised to blinded treatment with FP-1201-lyo/placebo. The patient was administered the first dose of study drug on 23 May 2017 and ended the treatment on 24 May 2017. The patient received 10 μg once daily (intravenously) of the blinded study drug for 1 day. His medical/surgical history included pulmonary arterial hypertension, coronary artery disease, hypercholesterolaemia, lymphoma, cerebrovascular accident, osteoarthritis, type 2 diabetes mellitus, arrhythmia supraventricular, sinus node dysfunction, benign prostatic hyperplasia, chronic renal insufficiency (from 2014) and cardiac insufficiency (hospitalized first time March 2014).

On 23 May 2017, the patient was hospitalised due to an aneurysm rupture and was operated

immediately. After the procedure, the patient was anuric, he also had severe hypotension and

needed high level noradrenaline support. On the first operative day the decision was to stop the intensive care treatment because of the poor prognosis due to the chronic renal insufficiency and severe cardiac insufficiency that the patient had already before the aneurysm rupture. The patient died on 24 May 2017 at 18:25 (Day 1 of the study). Ruptured abdominal aortic aneurysm was reported as a cause of death. An autopsy was not performed.

The investigator assessed the event of multiple organ dysfunction syndrome as not related to the study drug. The most likely cause of the event was concomitant disease. The sponsor agreed with the investigator’s causality assessment.

Patient 2

Patient 2, a 73-year-old male diagnosed with ruptured abdominal aortic aneurysm (RAAA),

was randomised to blinded treatment with FP-1201-lyo/placebo. The patient was administered the first dose of study drug on 04 Jun 2017 and ended the treatment prematurely on 05 Jun 2017. His medical/surgical history included type 2 diabetes mellitus, hypertension, acute myocardial infarction, cardiac arrest, ventricular fibrillation, sinus node dysfunction, hypercholesterolaemia, arrhythmia, cardiac failure congestive, ischaemic cardiomyopathy and hypothyroidism. On 04 Jun 2017, the patient sustained ruptured abdominal aortic aneurysm and was hospitalised. Emergency operation was carried out, bleeding during the operation was 12 litres. Postoperatively the patient was transferred to intensive care unit (ICU). He was anuric. Due to suspicion of bleeding and high intra-abdominal pressure, laparotomy and open abdomen treatment was initiated on 05 Jun 2017. Despite deliberation, the patient continued being anuric and hypotonic. Multi-organ failure was diagnosed considering its onset on 04 Jun 2017 at 18:30 (Day 0 of the study). The patient died on 05 Jun 2017 at 22:00 (Day 1 of the study). Ruptured abdominal aortic aneurysm was reported as the cause of death. An autopsy was not performed. The investigator assessed the event of multiple organ dysfunction syndrome as not related to the study drug. The most likely cause of the event was disease under treatment. The sponsor agreed with the investigator’s causality assessment.

**Supplemental Table S1. Reason for using glucocorticoids with IFN beta-1a**

| Reported indication for glucocorticoid use | Number of patients | Start of treatment | Dose & regime |
| --- | --- | --- | --- |
| Emphysema | 1 | 1 day after surgery | Hydrocortisone start 200mg/day, used tappering down until day 15 |
| Shock | 2 | From surgery | Hydrocortisone used for 2 days 200mg/day |
| Anesthesia | 1 | From surgery | Dexmethasone 9mg/day for 2 days |
| Baseline disease | 3 | Pre-existing | Methylprednisolon varying doses |


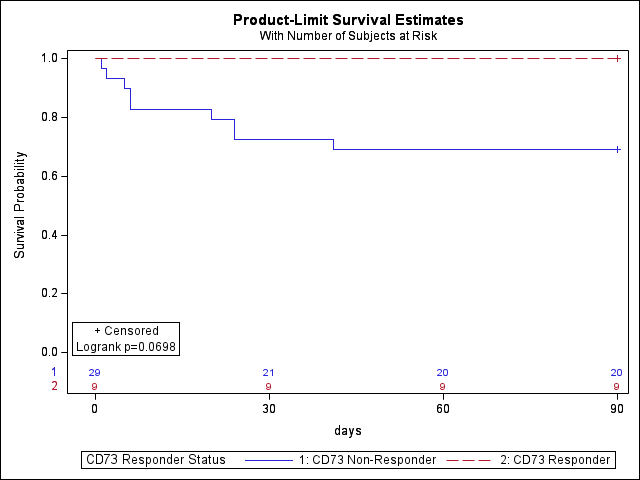


**Supplemental Figure S1. D90 Kaplan-Meier Survival Curve for the entire study population divided into CD73 responders vs. non-responders.**


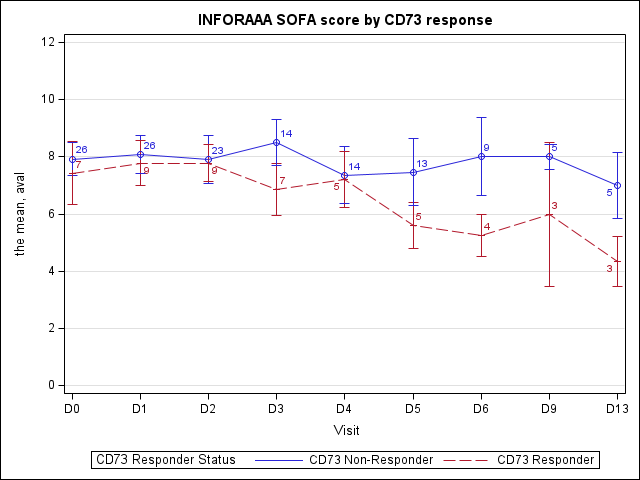


**Supplemental Figure S2. SOFA score in the entire study population divided into CD73 responders vs. non-responders**

**Methods for assessment of IFN beta neutralizing antibodies (NABS) and MxA and CD73 responses**

Blood samples for determination of biochemistry and haematology were taken at pre-dose on D0 (baseline value), D1, D2, D3, D4, D5, D6, D9, D13 and D30 (or on a particular date if a patient was discharged from initial operating hospital or discontinues from the study after D6 but prior to D30). Patient were not invited to laboratory visit, once discharged from hospital, except on D30 for assessment of NAbs. The date and time of collection was recorded in the eCRF.

The study of anti-human IFN beta antibodies in human serum was carried out in the quality-assured environment of Wieslab’s ISO 17025 and GLP test facility in Malmö, Sweden. The study was performed and documented according to current written local standard operating procedures A detailed description of the method can be shared under confidentiality. The principle is a bridging immunoassay with electrochemiluminescence (ECL) as detection method where anti-drug antibodies form a bridge with labelled drug material. The ECL label emit light in response to an electrical current that is initiated at the electrodes at the bottom of the plate. The method consists of three tiers: 1) a screening assay, where anti-drug antibodies are detected. 2) a confirmatory assay, where anti-drug antibody specificity is confirmed and 3) a titer assay, where anti-drug antibodies are semi-quantitated. Assay sample output is reported qualitatively (tier 1/2) and semi quantitatively (tier 3).

- Tier 1: Samples with a response ≥ SCP were further tested in confirmatory assay
- Tier 2: Samples with a response ≥ CCP were reported as positive
- Tier 3: The titer result was determined as the lowest dilution/concentration of the diluted sample that was detected ≥ SCP and was reported as absolute titer with minimum required dilution factored in.

Blank pooled serum was used as negative control (NC). NCs were stored at -60°C to -90°C. Positive controls (low- and high-quality controls (QCs)) were prepared by spiking blank pooled serum with the positive control antibody. QCs were prepared frozen at approximately -60°C to -90°C at least 12 hours prior to analysis. These run qualifying controls were included in all assay tiers and runs in at least replicate n = 2x2 wells at each concentration level. Study serum samples were transferred and stored at -60°C to -90°C until analyses. Study samples were analysed as duplicates in each analytical run. The %CV for the duplicate determination must be ≤25%, If a study sample does not meet acceptance criteria it must be reanalysed according to Wieslab procedures.

ELISA method was developed and validated for determination of CD73 in human serum samples. CD73 method lower limit of quantification (LLOQ) is 0.400 ng/mL serum and the upper limit of quantification (ULOQ) is 6.40 ng/mL serum. Serum samples were analyzed as duplicates. Calibrators and controls were prepared by spiking PBS with 0.5% of human albumin and 10 % of CD73 depleted serum with recombinant CD73.

MxA protein concentrations were measured from lysed whole blood samples using a previously described specific ELISA(1) and adjustments (2). Method LLOQ is 5.0 ng/mL blood lysate and the ULOQ is 40 ng/mL blood lysate. Samples were analyzed as duplicates. Determined values were for 20x diluted samples (50 μl of blood in 950 μl of hypotonic buffer). The factor 20 was used when reporting the values.
